# Liraglutide Inhibits Osteoclastogenesis and Improves Bone Loss by Downregulating Trem2 in Female Type 1 Diabetic Mice: Findings From Transcriptomics

**DOI:** 10.3389/fendo.2021.763646

**Published:** 2021-12-15

**Authors:** Jie Yu, Yan-Chuan Shi, Fan Ping, Wei Li, Hua-Bing Zhang, Shu-Li He, Yuan Zhao, Ling-Ling Xu, Yu-Xiu Li

**Affiliations:** ^1^ Key Laboratory of Endocrinology of National Health Commission, Department of Endocrinology, Peking Union Medical College Hospital, Chinese Academy of Medical Sciences & Peking Union Medical College, Beijing, China; ^2^ Group of Neuroendocrinology, Diabetes and Metabolism Division, Garvan Institute of Medical Research, St Vincent’s Hospital, Sydney, NSW, Australia; ^3^ Faculty of Medicine, UNSW, Sydney, NSW, Australia

**Keywords:** type 1 diabetes, bone loss, bone density, bone microarchitecture, osteoclastogenesis, Triggering receptor expressed on myeloid cells 2, liraglutide, transcriptomics

## Abstract

**Background:**

The mechanisms of bone fragility in type 1 diabetes (T1D) are not fully understood. Whether glucagon-like peptide-1 receptor (GLP-1R) agonists could improve bone quality in T1D context also remains elusive.

**Aims:**

We aimed to explore the possible mechanisms of bone loss in T1D and clarify whether liraglutide has effects on bone quality of T1D mice using transcriptomics.

**Methods:**

Female streptozotocin-induced diabetic C57BL/6J mice were randomly divided into four groups and received the following treatments daily for 8 weeks: saline as controls, insulin, liraglutide, and liraglutide combined with insulin. These groups were also compared with non-STZ-treated normal glucose tolerance (NGT) group. Trunk blood and bone tissues were collected for analysis. Three tibia from each of the NGT, saline-treated, and liraglutide-treated groups were randomly selected for transcriptomics.

**Results:**

Compared with NGT mice, saline-treated T1D mice manifested markedly hyperglycemia and weight loss, and micro-CT revealed significantly lower bone mineral density (BMD) and deficient microarchitectures in tibias. Eight weeks of treatment with liraglutide alone or combined with insulin rescued the decreased BMD and partly corrected the compromised trabecular microarchitectures. Transcriptomics analysis showed there were 789 differentially expressed genes mainly mapped to osteoclastogenesis and inflammation pathways. The RT-qPCR verified that the gene expression of *Trem2*, *Nfatc1*, *Trap*, and *Ctsk* were significantly increased in the tibia of T1D compared with those in the NGT group. Liraglutide treatment alone or combined with insulin could effectively suppress osteoclastogenesis by downregulating the gene expression of *Trem2*, *Nfatc1*, *Ctsk*, and *Trap*.

**Conclusions:**

Taken together, increased osteoclastogenesis with upregulated expression of *Trem2* played an important role in bone loss of T1D mice. Liraglutide provided protective effects on bone loss in T1D mice by suppressing osteoclastogenesis.

## Introduction

Type 1 diabetes (T1D) is characterized by autoimmune destruction of pancreatic islet β cells leading to severe hyperglycemia (1). Glucose metabolism disturbance can gradually lead to diabetes-related chronic complications. These complications include diabetic nephropathy, diabetic retinopathy, and diabetic neuropathy. In addition, skeletal fragility has also been associated with type 1 diabetes, which exhibited as deficits in bone mineral density (BMD) and bone microarchitectures compared with controls, leading to increased fracture risks (2, 3).

The increased bone fragility in type 1 diabetes has been attributed to complex and multifactorial pathophysiological mechanisms which are only partially understood. Hyperglycemia, hypoinsulinemia, accumulation of advanced glycation end products, and increased marrow adiposity lead to a decrease in bone formation, bone mineralization, and poor osteoblast activity, but the effects on osteoclasts are less studied and inconsistent (4).

Transcriptomics, one of the omics technologies, focuses on the RNA transcripts that are produced by the genome under specific circumstances or in a specific cell. Transcriptomics is a powerful tool to investigate the molecular mechanisms behind complex and multifactorial systemic diseases in an unbiased and comprehensive manner (5). Thus far, there is no study showing the transcriptomic analysis of T1D-associated osteopenia or osteoporosis.

Furthermore, certain antidiabetic medications might also affect fracture risk independent of their glucose-lowering effects. Glucagon-like peptide-1 receptor (GLP-1R) agonists as adjunctive therapies have been tested in T1D patients (6), and some individuals who are overweight or have detectable levels of C-peptide might benefit from those medications (6). GLP-1R agonists also exert protective effects on bone tissue *in vitro* and *in vivo* (7–9). Some clinical studies have found neutral effects of liragutide on bone, but there was also a few meta-analysis of 16–59 randomized controlled trials with 11,206–49,602 patients with T2DM which showed that compared with placebo and other antidiabetic drugs, liraglutide was associated with a significant reduction in the risk of bone fractures (10–13). However, clinical and experimental research data in the context of T1D are scarce. Till now, only one experimental research by Mansur et al. found that liraglutide treatment for 21 days in streptozotocin (STZ)-induced T1D mice significantly increased indicators such as bone maximum force and hardness but failed to improve trabecular and cortical microarchitectures (14). We hypothesized that GLP-1R agonists might provide beneficial effects on T1D-associated osteopenia or osteoporosis.

Thus, in this study, we intended to explore underlying molecular mechanisms of bone fragility in T1D mouse models using transcriptomics. Then, liraglutide was administrated alone or in combination with insulin to T1D mice to explore their effects on bone quality and possible mechanisms.

## Research Design and Methods

### Animals and Experimental Design

Ten- to 11-week-old female C57BL/6J mice were purchased from Beijing Huafukang Co., Ltd. (Beijing, China) and allowed to acclimate to the environment for 1 week. Then, mice were treated either with STZ to induce diabetes (150 mg/kg body weight once) or with vehicle (100 mM citrate, pH 4.2–4.5), by intraperitoneal (i.p.) injection (15).

Two weeks later, the vehicle-injected mice with normal glucose tolerance (NGT) (*n* = 8) were assigned to group 1 (referred as to NGT). At the same time, STZ-injected confirmed diabetic mice (random blood glucose ≥250 mg/dl) (16) were randomly assigned to four treatment groups for 8 weeks (*n* = 8 per group): group 2 treated with saline (referred to as T1D); group 3 (referred to as INS) treated with insulin by subcutaneous injection (insulin dose 10 units/kg body weight/day, as detemir insulin, Levemir^®^, Novo Nordisk, Denmark), which referred to the insulin dose used in the previous study (17); group 4 (referred to as Lira), treated with liraglutide by subcutaneous injection (liraglutide dose: 0.6 mg/kg/day, Novo Nordisk, Denmark); and group 5 (referred to as Lira+INS), treated with insulin (10 units/kg/day) and liraglutide (0.6 mg/kg/day). The doses of liraglutide used in previous studies ranged from 0.2 to 1 mg/kg, so we chose a relatively large dose of 0.6 mg/kg (16, 18, 19).

All mice were maintained in a 12-h light–dark cycle at 22°C and provided *ad libitum* access to water and food for 8 weeks. Body weight and pedal dorsal vein blood glucose *via* Accu-Chek compact glucometer (Roche) were measured weekly. Upon the completion of the experiments, mice were killed by isoflurane overdose followed by decapitation, and trunk blood and bone tissues were collected. All animal procedures were approved by the Institutional Animal Care and Use Committee (IACUC) at the Institute of Laboratory Animals Science, CAMS and PUMC and conducted according to the Laboratory Animal Management Regulations in China and adhered to the Guide for the Care and Use of Laboratory Animals published by the National Institutes of Health (NIH Publication No. 85-23, revised 2011). All animal studies complied with the ARRIVE guidelines.

### Assessment of Skeletal Microarchitecture

After euthanasia, the left tibia was harvested and stored at −80°C until analysis. For bone microarchitecture analyses, the mid-shaft and proximal metaphysis regions along the axis of the bone were scanned by Inveon MM micro-CT manufactured by Siemens (Berlin, Germany) at a voltage of 70 kV and a current of 400 μA, with an entire scan length of 1 cm in a spatial resolution of 35 μm used for animal experimental studies and reconstructed using the Inveon analysis workstation. Tibia trabecular bone analyses were performed from 0.5 mm distal to the growth plate, extending 1 mm toward the diaphysis and excluding the cortical bone. Cortical measurements were performed in a 1-mm length centered midway down the length of the bone. Trabecular volumetric bone mineral density (Tb.vBMD), bone volume fraction (BV/TV), trabecular thickness (Tb.Th), trabecular separation (Tb.Sp), trabecular number (Tb.N), cortical volumetric bone mineral density (Ct.vBMD), and cortical bone thickness (Ct.Th) were computed according to the instruction of the manufacturer.

### Bone Histomorphometry

After mice were sacrificed, left femurs were harvested, cleaned, fixed in 4% paraformaldehyde solution at 4°C for 10–24 h, and decalcified in 10% ethylenediamine tetraacetic acid disodium (EDTA-2Na, pH 7.2) for 5–7 days. The decalcified femurs were dehydrated, embedded in paraffin, sectioned at 5 μm, stained with H&E, and observed for histopathological changes using standard light microscopy. The 1-mm region of trabecular bone starting from 0.5 mm below the distal femoral growth plate was selected as the region of interest. Measurements of tissue area (T.Ar, mm^2^), trabecular bone area (Tb.Ar, mm^2^), and trabecular perimeter (Tb.Pm, mm) and visible adipocytes which were greater than 30 μm were obtained directly from the software Image Pro Plus 6.0 (Media Cybernetics, Rockville, MD, USA) (20, 21). Then, bone volume fraction (BV/TV, %), trabecular thickness (Tb.Th, mm), trabecular separation (Tb.Sp, mm), trabecular number (Tb.N, 1/mm), and marrow adipose number (1/mm^2^) were obtained using standard formulas shown in **Supplementary Table 1** (22).

### Biochemical Measurements

The serum levels of procollagen type 1 N-terminal propeptide (P1NP) and C-terminal telopeptides of type 1 collagen (CTX) and C-peptide were measured using commercially available enzyme-linked immunosorbent assay (ELISA) kits (Cloud-Clone Corp., Ltd., Wuhan, China) according to the instructions of the manufacturer.

### Transcriptomics Sequencing and Bioinformatics Analysis

Immediately after euthanasia, bone samples were cleaned of all muscle and connective tissues, snap frozen in liquid nitrogen, and stored at −80°C until RNA extraction. Three tibia tissues were selected from each of the following groups: NGT, T1D, and Lira groups for high-throughput RNA sequencing. In total, nine tibia tissues were used for the analysis. In brief, total RNA was extracted using TRIzol reagent (Invitrogen Co., USA) according to the protocol of the manufacturer. The RNA was then checked for purity and stability by gel electrophoresis, and the concentration was determined using the Agilent 2100 Bioanalyzer (Agilent Technologies, Inc.). The qualified total RNA was digested with DNase I, enriched using oligo(dT) magnetic beads, and then fragmented. Fragmented mRNA was added to random primers for cDNA synthesis and PCR reaction to obtain a single-stranded DNA library. The qualified library was formed into DNA nanospheres (DNB) by rolling circle replication and finally sequenced on the computer.

Clean reads were obtained and aligned to the reference genome of mice. Based on the alignment results, the expression level of each gene was calculated, and the samples were analyzed further in terms of difference, enrichment, and cluster analysis.

Differentially expressed genes (DEGs) were defined as having a fold change >2 and *Q*-value <0.05 using one-way analysis of variance (ANOVA). DEGs had to appear in all three mice in the group to be considered. The Gene Ontology (GO) platform (http://www.geneontology.org/) was used to perform functional enrichment analysis of the DEGs (23). The Kyoto Encyclopedia of Genes and Genomes (KEGG; http://www.genome.jp/kegg/pathway.html) was used to determine significant pathways associated with the DEGs (24). Pathways with *Q*-value thresholds of <0.05 were considered potential target pathways.

### Verification of Identified Genes Using Quantitative Reverse Transcription Polymerase Chain Reaction

Total RNA was extracted using TRIzol reagent and reverse-transcribed to cDNA using oligo(dT). Quantitative reverse transcription polymerase chain reaction (RT-qPCR) for target genes was performed by using a SYBR Green kit (Biotium, USA). PCR was carried out on an ABI 7700 system (Roche LightCycler^®^ 480II, Switzerland) using the following reaction conditions: 5 min at 95°C, followed by 45 cycles of 10 s at 95°C and 30 s at 60°C. All gene expression levels were normalized to β-actin expression. The primers are listed in **Table 1**.

**Table 1:** Primers used in our experiments.

Primer name	Sequence (5′ to 3′)	Number of bases	Product length (bp)
β-Actin	F: GAGATTACTGCTCTGGCTCCTA	22	150
	R: GGACTCATCGTACTCCTGCTTG	22	
*Trem2*	F: ACTTATGACGCCTTGAAGCACTGG	24	236
	R: CCTCGGAGACTCTGACACTGGTAG	24	
*Ctsk*	F: CAGTGTTGGTGGTGGGCTATGG	22	174
	R: TGGCTGGCTGGAATCACATCTTG	23	
*c-Fos*	F: GCTGCACTACTTACACGTCTTCCT	24	169
	R: GCTGCCTTGCCTTCTCTGACTG	22	
*Trap*	F: ACGGCTACTTGCGGTTTCACTATG	24	172
	R: AAGCAGGACTCTCGTGGTGTTCA	23	
*Nfatc1*	F: GGTGAGGCTGGTCTTCCGAGTT	22	139
	R: GCTGTCTGTGCTCTGCTTCTCC	22	
*Opg*	F: CGGAGAGTGAGGCAGGCTATT	21	135
	R: GCTGTGAGGAGAGGAAGGAAGG	22	
*Rankl*	F: CATCGGGTTCCCATAAAG	18	141
	R: GAAAGCAAATGTTGGCGTA	19	
*Tnfa*	F: TAACTTAGAAAGGGGATTATGGCT	24	264
	R: TGGAAAGGTCTGAAGGTAGGAA	22	

### Statistical Analysis

Continuous variables were expressed as mean ± standard deviations (SDs); one-way ANOVA was used for comparison between groups, and the least significant difference (LSD) method was used for multiple comparisons. Two-tailed tests were used for all statistics, and *p <*0.05 was defined as statistically significant differences. Statistical analysis of all data was performed in SPSS 25.0 software (SPSS Inc., Chicago, IL, USA). All figures were performed in GraphPad Prism 8.0 software (GraphPad, La Jolla, USA).

## Results

### Effects of Liraglutide on Body Weight and Glucose Control in T1D Mice

**Figure 1:**
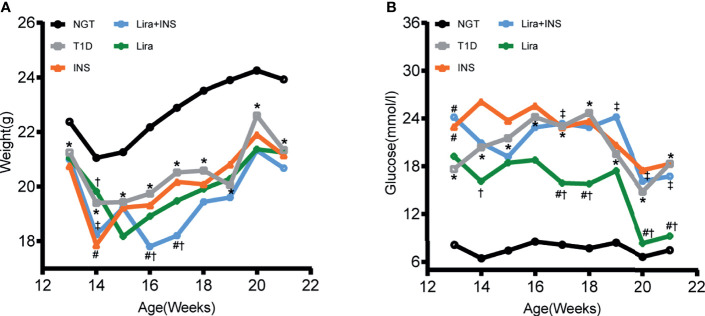
Changes of body weight and blood glucose during treatments. **(A)** Weekly body weight (g); **(B)** weekly blood glucose levels at fed states (mmol/l). NGT: normal glucose tolerance group; T1D, saline-treated type 1 diabetes group; INS, insulin treatment group; Lira, liraglutide treatment group; INS+Lira, insulin + liraglutide treatment group; ANOVA was used for comparison between groups, and *p <*0.05 was defined as statistically significant. *compared with NGT; ^#^compared with T1D; ^†^compared with INS; ^‡^compared with Lira. All data are expressed as mean ± SDs.

As expected, saline-treated T1D mice manifested markedly hyperglycemia and weight loss when compared with NGT controls, confirming the successful establishment of the disease model (**Figure 1** and **Supplementary Tables 2**, **3**). However, in comparison with saline-treated T1D mice, once-daily insulin detemir treatment failed to control blood sugar well or recover weight loss but reduced deaths due to hyperglycemia (**Figure 1** and **Supplementary Tables 2**, **3**). Furthermore, liraglutide monotherapy significantly improved glucose control especially in the second half of the experiment compared with the saline-treated T1D group and insulin treatment group but did not restore weight loss, probably because liraglutide partly restored β-cell function but also had weight loss effect (**Figure 1** and **Supplementary Tables 2**, **3**). However, the combined treatment with liraglutide and insulin did not improve glycemic control and even caused further weight loss compared with saline-treated T1D mice (**Figure 1** and **Supplementary Tables 2**, **3**).

### Effects of Liraglutide on Bone Mineral Density and Microarchitectures

**Figure 2:**
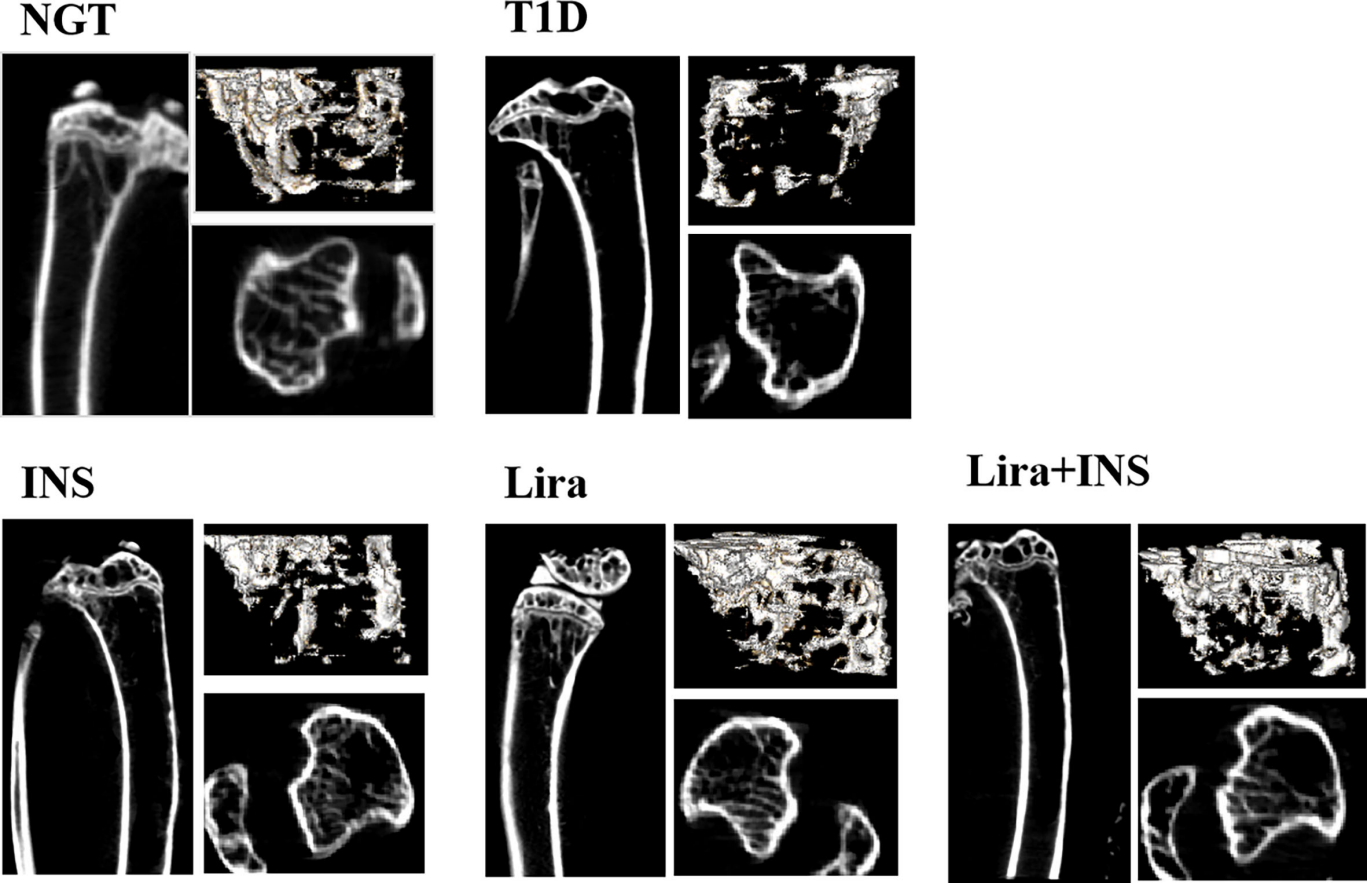
Typical 2D and 3D images of micro-CT. NGT, normal glucose tolerance group; T1D, type 1 diabetes group; INS, insulin treatment group; Lira, liraglutide treatment group; INS+Lira, insulin + liraglutide treatment group.

**Figure 3:**
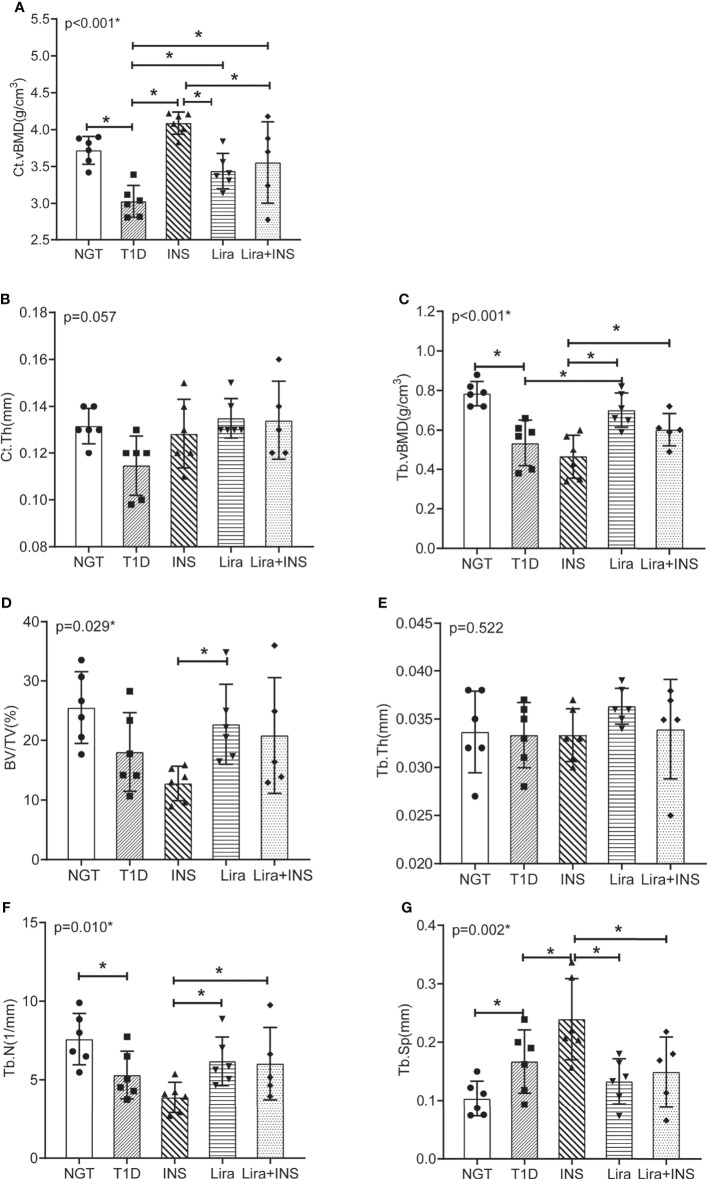
Bone mineral density and microarchitectures of tibia measured by micro-CT. **(A)** Cortical volumetric bone mineral density (Ct.vBMD); **(B)** cortical bone thickness (Ct.Th); **(C)** trabecular volumetric bone mineral density (Tb.vBMD); **(D)** bone volume fraction (BV/TV); **(E)** trabecular thickness (Tb.Th); **(F)** trabecular number (Tb.N); **(G)** trabecular separation (Tb.Sp). NGT, normal glucose tolerance group; T1D, type 1 diabetes group; INS, insulin treatment group; Lira, liraglutide treatment group; INS+Lira, insulin + liraglutide treatment group. ANOVA was used for comparison between groups, and *p <* 0.05 was defined as statistically significant. *was used to indicate statistical difference. All data are expressed as mean ± SDs.

In the tibia, saline-treated T1D mice showed reduction in cortical volumetric bone mineral density (Ct.vBMD) and cortical thickness (Ct.Th) compared with NGT controls, while the difference in cortical thickness did not reach statistical significance (**Figures 2**, **3** and **Supplementary Table 4**). In addition, deficits in trabecular bone of the tibia were also apparent; especially, reductions in trabecular number (Tb.N) and trabecular volumetric bone mineral density (Tb.vBMD), along with an increase in trabecular separation, were significant in T1D mice in relation to NGT controls (**Figures 2**, **3** and **Supplementary Table 4**).

Compared with the T1D group, treatment with insulin alone rectified the decreased Ct.vBMD, but seemed to further deteriorate the trabecular microarchitectures, especially significantly increased Tb.Sp of the tibia (**Figures 2**, **3** and **Supplementary Table 4**). However, when treated with liraglutide alone or combined with insulin, significant recovery of the tibia Tb.vBMD and Ct.vBMD, along with partially recovery of tibia trabecular microarchitectures, was evident in mice receiving monotherapy or combined therapy (**Figures 2**, **3** and **Supplementary Table 4**).

### Effects of Liraglutide on Bone Histomorphometry

**Figure 4:**
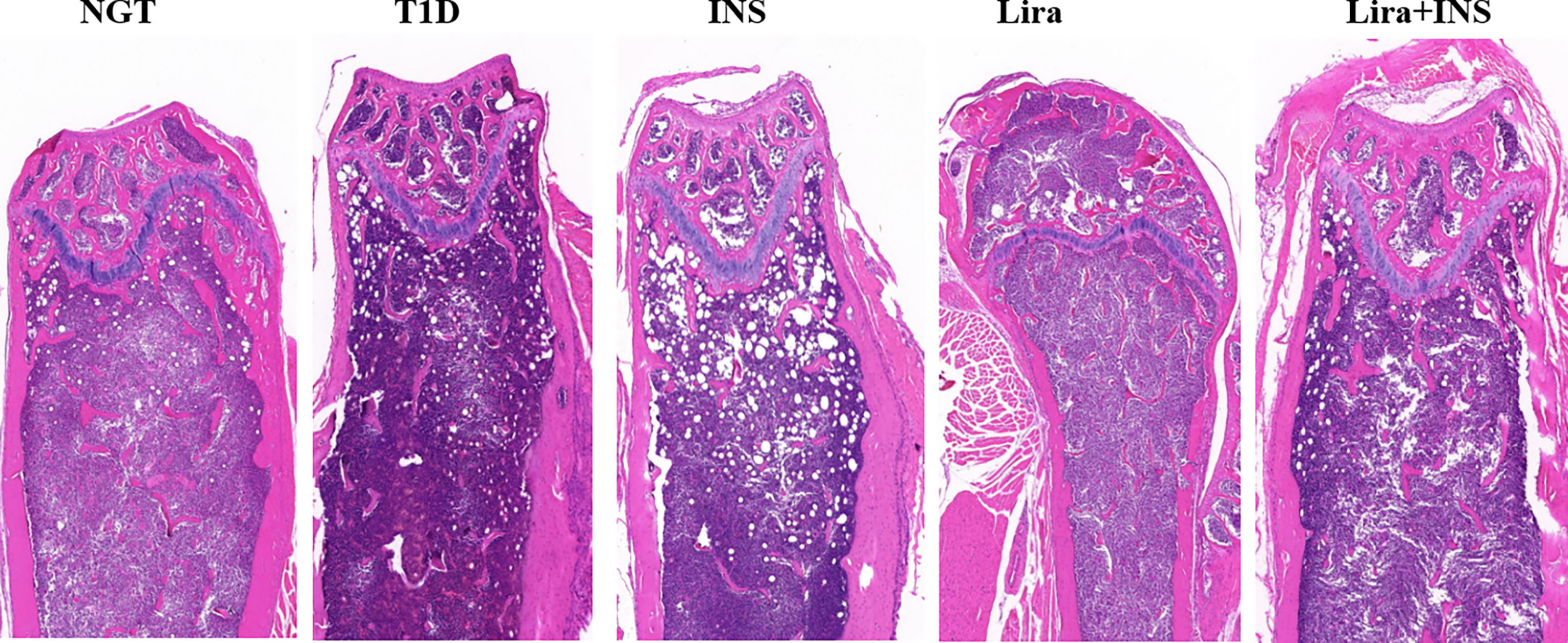
Typical H&E images of bone histomorphometry. NGT, normal glucose tolerance group; T1D, type 1 diabetes group; INS, insulin treatment group; Lira, liraglutide treatment group; INS+Lira, insulin + liraglutide treatment group.

**Figure 5:**
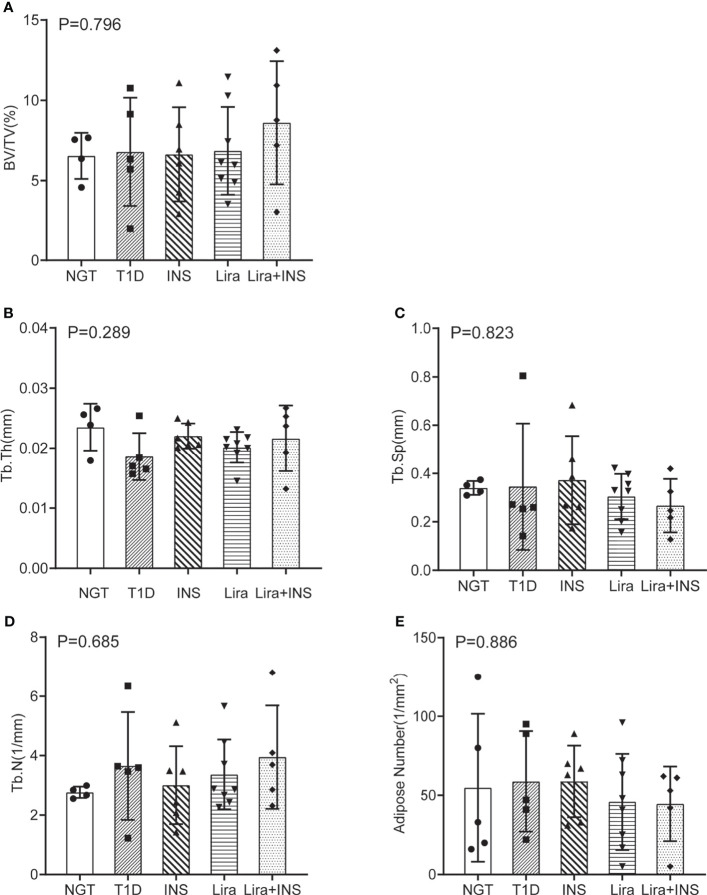
Bone histomorphometry of the femur. **(A)** Bone volume fraction (BV/TV); **(B)** trabecular thickness (Tb.Th); **(C)** trabecular separation (Tb.Sp); **(D)** trabecular number (Tb.N); **(E)** adipose number in marrow. NGT, normal glucose tolerance group; T1D, type 1 diabetes group; INS, insulin treatment group; Lira, liraglutide treatment group; INS+Lira, insulin + liraglutide treatment group. ANOVA was used for comparison between groups, and *p <* 0.05 was defined as statistically significant. All data are expressed as mean ± SDs.

As shown in typical pictures of femur histomorphometry, saline-treated T1D mice showed thinner and broken trabeculae, increased trabecular spacing, and more adipocytes in bone marrow. Liragutide treatment alone or combined with insulin partly improved those defects (**Figure 4**). However, quantitative analysis did not demonstrate significance between group differences for BV/TV, Tb.Th, Tb.N, Tb.Sp, and marrow adipose number (**Figure 5** and **Supplementary Table 5**).

### Effects of Liraglutide on Bone Turnover and C-Peptide

**Figure 6:**
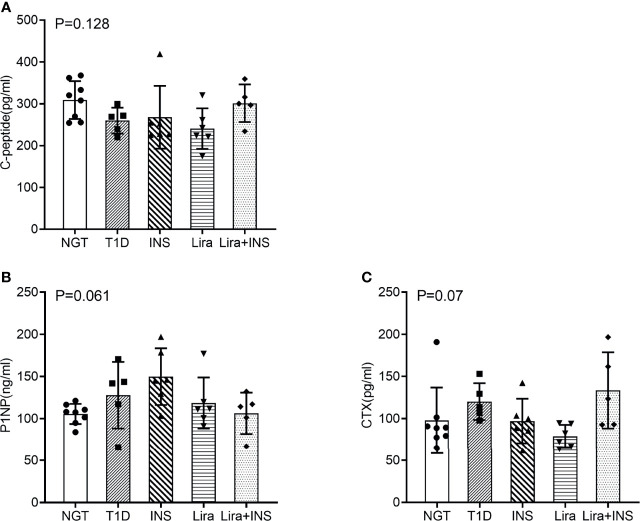
Serum bone turnover markers and C-peptide. **(A)** C-peptide; **(B)** procollagen type 1 N-terminal propeptide (P1NP); **(C)** C-terminal cross-linking telopeptide of type 1 collagen (CTX). NGT, normal glucose tolerance group; T1D, type 1 diabetes group; INS, insulin treatment group; Lira, liraglutide treatment group; INS+Lira, insulin + liraglutide treatment group; ANOVA was used for comparison between groups, and *p <* 0.05 was defined as statistically significant. All data are expressed as mean ± SDs.

Bone turnover markers in serum were then detected. P1NP is a marker of bone formation, and CTX is a marker of bone resorption. P1NP and CTX both showed non-significant trends toward an increased level in T1D mice compared with NGT mice (**Figure 6**, **Supplementary Table 6**). Insulin treatment slightly increased P1NP and decreased CTX, liraglutide treatment alone decreased CTX more than PINP, while liraglutide combined with insulin treatment decreased PINP and increased CTX, but all these comparisons did not reach significance (**Figures 6B, C** and **Supplementary Table 6**). The CTX in the liraglutide +insulin treatment group seemed increased; however, the sample in this group was relatively small and might be greatly affected by extreme values.

Serum C-peptide was detected using the serum obtained at the end of the experiment. There was no significant difference among each group (**Figure 6A**). These results were inconsistent with the blood glucose, and we found that C-peptide levels in the insulin treatment groups were higher than those in the other groups. Therefore, we speculated that there might be cross-reactions with insulin during the C-peptide detection.

### Transcriptome Analysis of DEGs Between the NGT, T1D, and Liraglutide Treatment Groups

To further explore the mechanisms of diabetic-associated bone loss, we selected three tibia specimens from each of the NGT, T1D, and liraglutide treatment groups for transcriptome sequencing and bioinformatics analysis.

**Figure 7:**
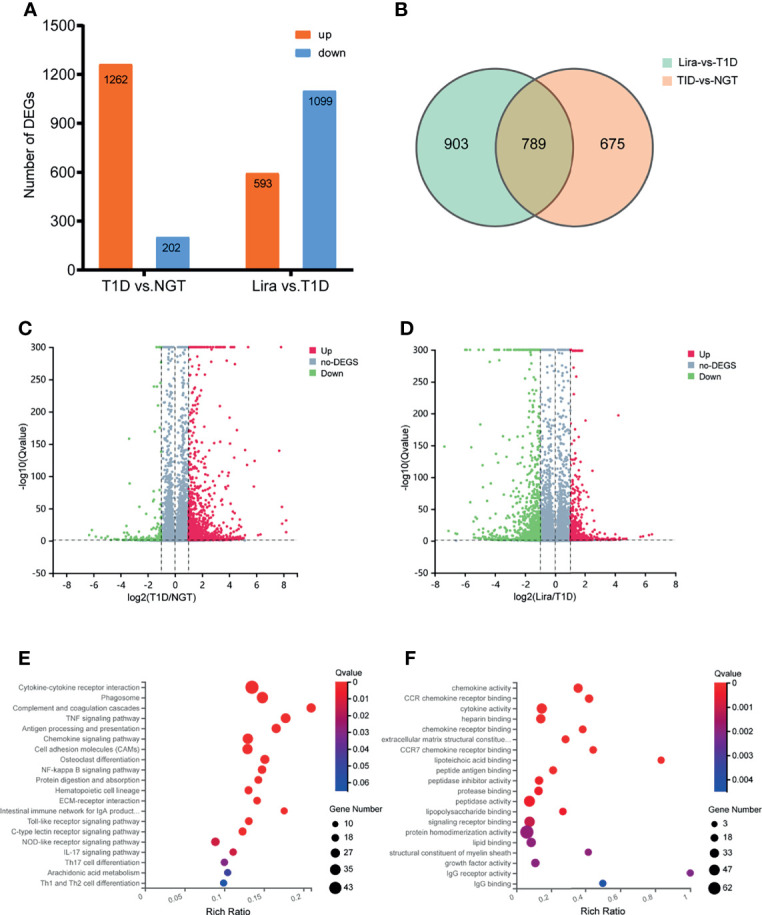
Transcriptomic analysis of DEGs between NGT, T1D, and liraglutide treatment groups. **(A)** Number of DEGs in histogram; **(B)** Venn diagram of pairwise comparison; **(C)** volcano map of DEGs between the NGT and T1D groups; **(D)** volcano map of DEGs between the T1D and liraglutide groups. **(E)** Bubble plots using the KEGG database; **(F)** bubble plots using the GO-MF database. NGT, normal glucose tolerance group; T1D, type 1 diabetes group; Lira, liraglutide treatment group.

Transcripts of DEGs were characterized using RNA-sequencing (RNA-seq) analyses of tibia tissues from the NGT, T1D, and liraglutide treatment groups. A fold change >2 and *Q*-value <0.05 were used to screen the DEGs. The results revealed obvious differences between the NGT group and the T1D group and between the T1D group and the liraglutide treatment group, which are presented in histogram and volcano map (**Figures 7A, C, D**). Compared with the NGT group, 1,464 genes were significantly changed in the tibia tissues in T1D mice consisting of 1,262 upregulated and 202 downregulated genes. On the other hand, compared with the untreated T1D group, 1,692 genes were significantly changed in the tibia tissues in liraglutide treatment mice consisting of 593 upregulated and 1,099 downregulated genes. A total of 789 DEGs were shared by these two comparisons (**Figure 7B**).

Then, GO and KEGG databases were used to annotate and classify those 789 shared DEGs. The enrichment analysis indicated that those DEGs were mainly distributed in the signals such as osteoclast differentiation (osteoclastogenesis) signaling pathway, tumor necrosis factor (TNFa) signaling pathway, and nuclear factor-κB (NF-κB) signaling pathway (according to the KEGG database), and the functional enrichment was mainly concentrated in processes, such as chemokine activity and cytokine activity (according to the GO-MF database) (**Figures 7E, F** and **Tables 2**, **3**). Thus, the increased osteoclastogenesis and enhanced inflammation were the characteristic biological changes in tibia bone remodeling in diabetic bone, and liraglutide treatment might inhibit osteoclastogenesis and inflammation. Significantly enriched DEGs in the osteoclastogenesis pathway are manifested in **Table 4**, and the fold change of Triggering receptor expressed on myeloid cells 2 (*Trem2*) gene expression ranked among the top (**Table 4**).

**Table 2:** Significantly enriched pathways of DEGs using the KEGG database.

ID	Description	Gene number	Rich ratio	*Q*-value
4060	Cytokine–cytokine receptor interaction	43	0.135	<0.001
4145	Phagosome	33	0.148	<0.001
4610	Complement and coagulation cascades	22	0.210	<0.001
4668	TNF signaling pathway	25	0.177	<0.001
4612	Antigen processing and presentation	22	0.165	<0.001
4062	Chemokine signaling pathway	28	0.130	<0.001
4514	Cell adhesion molecules (CAMs)	27	0.129	<0.001
4380	Osteoclast differentiation	21	0.151	<0.001
4064	NF-kappa B signaling pathway	18	0.148	<0.001
4974	Protein digestion and absorption	14	0.143	0.001
4640	Hematopoietic cell lineage	15	0.130	0.001
4512	ECM–receptor interaction	13	0.141	0.001
4672	Intestinal immune network for IgA production	10	0.175	0.001
4620	Toll-like receptor signaling pathway	14	0.131	0.001
4625	C-type lectin receptor signaling pathway	15	0.123	0.001
4621	NOD-like receptor signaling pathway	18	0.089	0.013
4657	IL-17 signaling pathway	12	0.111	0.013
4659	Th17 cell differentiation	12	0.100	0.031
590	Arachidonic acid metabolism	10	0.104	0.048
4658	Th1 and Th2 cell differentiation	10	0.099	0.065

**Table 3:** Significantly enriched processes of DEGs using the GO-MF database.

GO_F term ID	Description	Gene number	Rich ratio	*Q*-value
GO:0008009	Chemokine activity	24	0.358	<0.001
GO:0048020	CCR chemokine receptor binding	16	0.421	<0.001
GO:0005125	Cytokine activity	34	0.145	<0.001
GO:0008201	Heparin binding	25	0.140	<0.001
GO:0042379	Chemokine receptor binding	10	0.385	<0.001
GO:0030020	Extracellular matrix structural constituent conferring tensile strength	11	0.282	<0.001
GO:0031732	CCR7 chemokine receptor binding	8	0.444	<0.001
GO:0070891	Lipoteichoic acid binding	5	0.833	<0.001
GO:0042605	Peptide antigen binding	12	0.211	<0.001
GO:0030414	Peptidase inhibitor activity	17	0.130	<0.001
GO:0002020	Protease binding	17	0.127	<0.001
GO:0008233	Peptidase activity	41	0.074	<0.001
GO:0001530	Lipopolysaccharide binding	8	0.267	<0.001
GO:0005102	Signaling receptor binding	36	0.075	0.001
GO:0042803	Protein homodimerization activity	62	0.060	0.002
GO:0008289	Lipid binding	26	0.084	0.002
GO:0019911	Structural constituent of myelin sheath	5	0.417	0.002
GO:0008083	Growth factor activity	17	0.108	0.002
GO:0019770	IgG receptor activity	3	1.000	0.002
GO:0019864	IgG binding	4	0.500	0.004

**Table 4:** Significantly enriched DEGs in the osteoclastogenesis pathway.

Gene ID	Gene symbol	log2 (T1D/NGT)	*Q*-value (T1D/NGT)
15978	“Ifng”	4.545	0.00
246256	“Fcgr4”	3.904	<0.001
83433	“Trem2”	3.722	<0.001
14129	“Fcgr1”	3.397	<0.001
435653	“Fcrlb”	2.932	0.001
320832	“Sirpb1a”	2.906	<0.001
100038947	“LOC100038947”	2.714	<0.001
18729	“Pira6”	2.336	<0.001
13038	“Ctsk”	2.335	<0.001
14130	“Fcgr2b”	2.176	<0.001
11433	“Acp5”	2.163	<0.001
14131	“Fcgr3”	2.160	<0.001
21934	“Tnfrsf11a”	2.059	<0.001
12703	“Socs1”	2.001	<0.001
232790	“Oscar”	1.947	<0.001
18722	“Pira1”	1.825	<0.001
12978	“Csf1r”	1.825	<0.001
20846	“Stat1”	1.820	<0.001
12702	“Socs3”	1.705	<0.001
14200	“Fhl2”	1.419	<0.001
21943	“Tnfsf11”	1.281	0.002

### RT-qPCR Verification of Differentially Expressed Genes Identified by RNA-Seq

To validate transcriptomics analysis results, we analyzed the mRNA expression levels of representative genes using RT-qPCR. The selected genes were mainly in the signaling pathways of osteoclastogenesis and TNFa inflammation, including osteoprotegerin (*Opg*), nuclear factor receptor activator κB ligand (*Rankl*), *Tnfa*, *Trem2*, *c-Fos*, tartrate-resistant acid phosphatase (*Trap*), cathepsin K (*Ctsk*), and nuclear factor-activated T cell 1 (*Nfatc1*) (**Table 4**).

**Figure 8:**
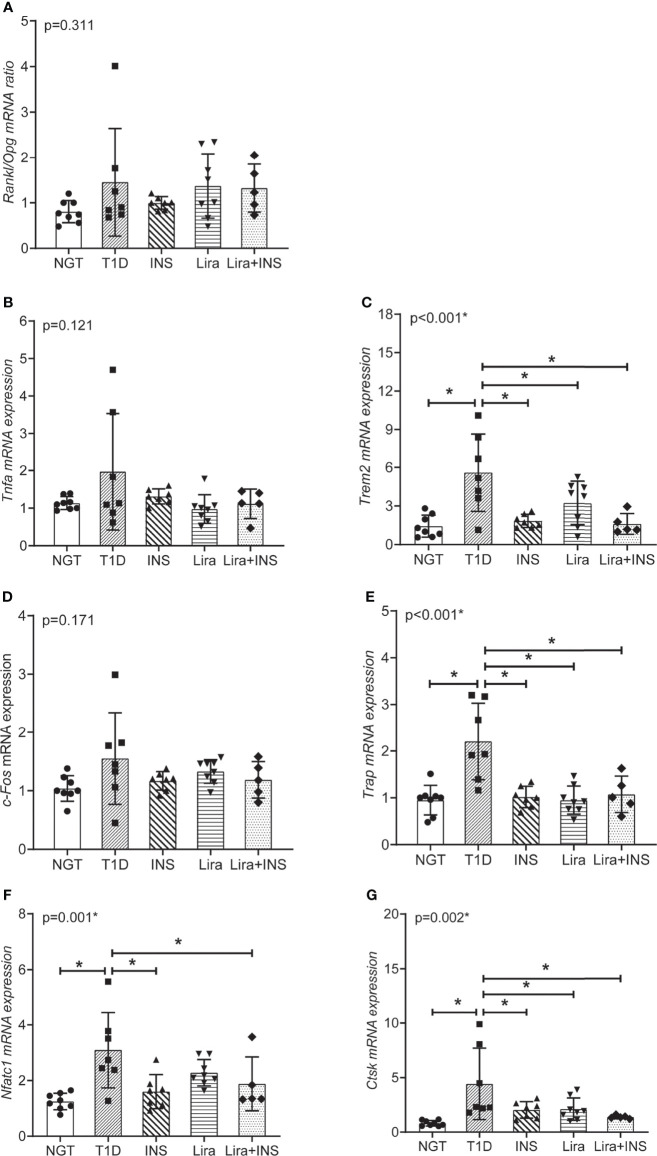
Relative mRNA expression in the signaling pathways of osteoclastogenesis and TNFa inflammation in tibia tissues. **(A)** The ratio of *Rankl*/*Opg* mRNA expression; **(B)** *Tnfa* mRNA expression; **(C)** *Trem2* mRNA expression; **(D)** *c-Fos* mRNA expression; **(E)** *Trap* mRNA expression; **(F)** *Nfatc1* mRNA expression; **(G)** *Ctsk* mRNA expression. NGT, normal glucose tolerance group; T1D, type 1 diabetes group; Lira, liraglutide treatment group; INS+Lira, insulin + liraglutide treatment group. ANOVA was used for comparison between groups, and *p <* 0.05 was defined as statistically significant. * was used to indicate statistical difference. All data are expressed as mean ± SDs.

The mRNA expression of genes expressed in the late phase of osteoclastogenesis including *Nfatc1*, *Trap*, and *Ctsk* were significantly increased in T1D mice compared with NGT except c-Fos, indicating enhanced osteoclast activity in diabetic bone (**Figures 8D**–**G**). The ratio of Rankl/Opg mRNA, a key regulator of osteoclastogenesis, only showed non-significant trends toward an increased level in T1D mice compared with NGT mice. However, the mRNA level of *Trem2*, a key co-stimulatory molecular of osteoclastogenesis, was significantly increased in T1D mice compared with that in NGT mice (**Figures 8A, C**). Tnfa mRNA, an inflammation marker, also increased in T1D mice compared with that in NGT mice, but did not reach significance (**Figure 8B**).

Compared with untreated T1D mice, treatment with insulin alone, liraglutide alone, or liraglutide combined with insulin all significantly decreased *Trem2*, *Nfatc1*, *Trap*, and *Ctsk* expression, indicating their role in inhibiting osteoclastogenesis (**Figure 8**).

## Discussion

In this study, our main findings were as follows: 1) compared with the NGT group, T1D mice showed decreased BMD and compromised microarchitectures. Insulin treatment alone only rectified the decreased Ct.vBMD but seemed to further deteriorate the trabecular microarchitectures. However, liraglutide treatment alone or combined with insulin significantly recovered the tibia Tb.vBMD and Ct.vBMD and partially recovered the tibia trabecular microarchitectures. 2) Transcriptome analysis showed enhanced osteoclastogenesis and increased inflammation in tibia bone remodeling in diabetic bone. Further RT-qPCR verified that osteoclastogenesis was prominently increased in T1D mice, including upregulated expression of *Trem2, Nfatc1, Trap* and *Ctsk*, which could be inhibited by liraglutide treatment alone or combined with insulin.

In terms of bone turnover markers, we found no significant intergroup differences overall. Compared with bone histomorphology or local gene expression, serum turnover markers may be less sensitive. In our study and other studies, serum CTX or P1NP in T1D patients was within the normal range or similar to the control groups, while micro-CT found significant compromised microarchitectures (3, 25). Previous studies have found low bone turnover especially reduced bone formation in the T1D context (26); however, the bone resorption can be enhanced, inhibited, or unaltered, which might be attributed to the timing of onset of T1D, the duration of diabetes, and the disease-associated inflammatory environment (27). In our study, STZ-induced T1D mice showed significantly elevated blood glucose for an enough duration (at least 8 weeks), resulting in decreased BMD and compromised microarchitectures, which was consistent with previous clinical and animal studies (3, 14, 25, 28), indicating our successful establishment of the disease model. Thus, the STZ-induced T1D model could be a convenient and reliable model for studying T1D-related bone loss.

Furthermore, bone fragility of T1D is attributed to complex and multifactorial pathophysiological mechanisms, but much remained unknown. In order to have a more comprehensive and unbiased understanding of pathophysiologic mechanisms behind the bone fragility in T1D, we conducted transcriptomics and bioinformatics analyses, which have not been carried out in this setting till now. The transcriptomics in our study showed that there were a total of 789 DEGs including upregulated or downregulated genes, which were mainly mapped to osteoclastogenesis and inflammation pathways. A previous study using microarray analysis in a fracture healing mouse model revealed an upregulation of gene sets related to inflammation and elevated osteoclast numbers in the type 1 diabetic group (29), which was consistent with our results. On the other hand, in the distraction osteogenesis model, microarray analysis also found that T1D negatively affected the expression of transcription factors regulating osteoblast differentiation (30). Transcriptomic findings were only reported in a guided bone regeneration model under T1D circumstance and a differential expression of genes associated with the ossification process was evident at 15 days of healing between the healthy controls and diabetic animals (31). Different animal models and experimental conditions might lead to those inconsistent results.

RT-qPCR verified some key findings obtained from the transcriptome analysis. Previously, studies about T1D effects on osteoclasts most focused on gene expression of *Rankl/Opg* ratio, *Trap*, or *Cstk*. TRAP and CSTK, which are both important enzymes expressing in the late phase of osteoclastogenesis, have been found to increase in T1D, supporting our results (32). However, studies about RANKL/OPG ratio in T1D, the key regulator of osteoclastogenesis, have not been consistent, suggesting that RANKL/RANK-induced osteoclast activation might not be the primary mechanism that drives bone resorption throughout the course of T1D (27). In our study, RANKL/OPG ratio was not different between the T1D group and NGT mice, indicating that there might be alternative mechanisms in regulating osteoclastogenesis. Indeed, we have identified two significant DEGs in osteoclastogenesis pathways which have not been reported previously: *Nfatc1* and *Trem2*. *NFATc1* is the important transcription factor of osteoclastogenesis, while *Trem2* is upstream of *NFATc1* and is the key co-stimulatory molecule of osteoclastogenesis. *In vivo* studies have shown that mutations in *Trem2* have been associated with Nasu–Hakola disease that is characterized by cystic bone lesions, osteoporotic features, and loss of white matter in the brain (33). *In vitro* evidence suggests that the dysfunction of *Trem2* leads to significantly impaired osteoclastogenesis (34). In the present study, the *Trem2* gene ranked among the top of DEGs in the osteoclastogenesis pathway, suggesting its important role in regulating osteoclastogenesis in bone in the T1D context.

In this study, we also explored whether hypoglycemic therapy improved bone loss in T1D mice. Insulin is the primary therapy for T1D and two previous studies found that systemic insulin therapy could rescue inhibited osteogenesis and correct distorted bone microstructures (30, 35). In our study, insulin treatment alone was found to inhibit the gene expression of osteoclastogenesis and rectified the decreased Ct.vBMD, but paradoxically seemed to further deteriorate trabecular microarchitectures. The exact mechanisms that cause the discrepancy are not clear. In the two previous studies, insulin implants were used to achieve optimal glucose control, but in our study, once-daily insulin detemir treatment failed to control glucose well and caused large glucose fluctuations. Irwin et al. found that bone loss in T1D was caused by hyperglycemia more than the lack of insulin signaling in bone (36), which might explain our negative results of insulin treatment.

GLP-1R agonists have demonstrated some benefits as adjunct therapies in T1D patients (6). The only experimental research by Mansur et al. found that liraglutide treatment for 21 days in STZ-induced T1D mice significantly increased bone maximum force and hardness but failed to improve trabecular and cortical microarchitectures (14). In our study, we found that liraglutide treatment alone or in combination with insulin for 8 weeks not only rectified the decreased trabecular and cortical BMD, but also partly rectified the trabecular microarchitectures, which might be due to the longer treatment duration than the study of Mansur and colleagues. Moreover, mechanical forces are associated with proper skeletal homeostasis (37). Bone loss occurs in humans after immobilization and in animals subjected to tail suspension, in which case sclerostin is increased to mainly inhibit bone formation (37). Studies also found that weight loss is generally associated with decreased bone mass in humans and rodents accompanied by increased bone resorption and decreased bone formation (38, 39). In our study, the mice in the liraglutide treatment groups suffered greater weight loss than the T1D mice which might cause negative effect on bone, which might partly explain the only partial improvement of bone microarchitectures. Metformin was also found to have an effect on bone metabolism, which was also mainly associated with neutral outcomes or decreased fracture risk in comparison to treatment with other glucose-lowering drugs (10). Metformin was found to decrease ALP, CTX-1, TRACP 5b, PINP, and RANKL, but increase OPG, RUNX2, and OPG/RANKL ratio in diabetic rat, indicating a bone protection role (40), but it was not exactly the same as liraglutide.

As for the mechanisms, previous studies found that Glp1-r KO mouse manifested an increased number of osteoclasts and eroded surfaces (7) and GLP-1RAs could decrease osteoclastic surfaces (41, 42), indicating a control of bone resorption. However, the downstream molecular mechanisms underlying this effect have not ever been identified. Yamada et al. once proposed an indirect effect through a reduction in calcitonin gene expression in GLP-1r-deficient animals. In our study, liraglutide treatment alone or in combination with insulin could not only effectively suppress osteoclastogenesis by downregulating the expression of Trem2 and NFATc1, but also downregulated the expression of CTSK and TRAP to inhibit the resorptive activity, confirming its effect on bone resorption. Liraglutide was also found to inhibit osteoclastogenesis *in vitro*, which supported our results (43). However, due to the scarcity of research in this area, more evidence may be required to further clarify its role in osteoclastogenesis.

Our research has some limitations: although dynamic bone formation rate and bone resorption surface were not calculated, detailed micro-CT analysis and serum bone turnover indicators could also reflect the bone turnover process well; in addition, due to the small amount of serum in mice, serum TREM2 protein was not measured.

## Conclusion

In our study, T1D mice were confirmed to show decreased BMD and compromised microarchitectures. Transcriptomics was innovatively used to clarify pathophysiologic mechanisms, and we found that strengthened osteoclastogenesis regulated by increased expression of *Trem2* and downstream genes played an important role in type 1 diabetic bone phenotype. Moreover, liraglutide generated a bone-protective effect in T1D mice by suppressing osteoclastogenesis to inhibit bone resorption.

## Data Availability Statement

The data presented in the study are deposited in the Gene Expression Omnibus (GEO) repository, accession number GSE189112.

## Ethics Statement

The animal study was reviewed and approved by the Institutional Animal Care and Use Committee (IACUC) at the Institute of Laboratory Animal Sciences, CAMS and PUMC.

## Author Contributions

JY: animal experiment, data acquisition, and drafting of the manuscript; FP, HZ, WL, SH, and YZ: data acquisition and analysis and interpretation of the data. YS, LX, and YL: study concept and design, critical revision of the manuscript for important intellectual content, and study supervision. All authors contributed to the article and approved the submitted version.

## Funding

This study was supported by grants from the National Key Research and Development Program of China (Grant No.2016YFC1305000), China Diabetes Young Scientific Research Project (Grant No.2018-N-01), Non-profit Central Research Institute Fund of Chinese Academy of Medical Sciences (Grant No.2019XK320031), National Natural Science Foundation of China (Grant No.82100947), and the Science and Technology Base and Talent Project of the Guangxi Zhuang Autonomous Region, China (AD19259001).

## Conflict of Interest

The authors declare that the research was conducted in the absence of any commercial or financial relationships that could be construed as a potential conflict of interest.

## Publisher’s Note

All claims expressed in this article are solely those of the authors and do not necessarily represent those of their affiliated organizations, or those of the publisher, the editors and the reviewers. Any product that may be evaluated in this article, or claim that may be made by its manufacturer, is not guaranteed or endorsed by the publisher.
