# Supplementary material for: Liraglutide Inhibits Osteoclastogenesis and Improves Bone Loss by Downregulating Trem2 in Female Type 1 Diabetic Mice: Findings From Transcriptomics
Source: Front Endocrinol (Lausanne). 2021 Dec 15;12:763646. doi: 10.3389/fendo.2021.763646 (PMC8715718; doi:10.3389/fendo.2021.763646)
Supplement: Supplementary file 1 [file Table_1.docx]

**Supplementary table 1** Indirect parameters of trabecular bone calculated by formulas

| Parameters | Formulas | Units |
| --- | --- | --- |
| BV/TV | (Tb.Ar/T.Ar)×100 | % |
| Tb.Th | (2/1.199)*(Tb.Ar/Tb.Pm) | mm |
| Tb.Sp | (2/1.199)*(T.Ar-Tb.Ar)/Tb.Pm | mm |
| Tb.N | (1.199/2)*(Tb.Pm/T.Ar) | 1/mm |
| Marrow Adipose | adipose number/T.Ar | 1/mm^2^ |

Tissue area(T.Ar),Trabecular bone area(Tb.Ar),Trabecular perimeter(Tb.Pm).

Bone volume fraction (BV/TV),Trabecular thickness (Tb.Th), Trabecular separation (Tb.Sp), Trabecular number (Tb.N).
