# Supplementary material for: Liraglutide Inhibits Osteoclastogenesis and Improves Bone Loss by Downregulating Trem2 in Female Type 1 Diabetic Mice: Findings From Transcriptomics
Source: Front Endocrinol (Lausanne). 2021 Dec 15;12:763646. doi: 10.3389/fendo.2021.763646 (PMC8715718; doi:10.3389/fendo.2021.763646)
Supplement: Supplementary file 2 [file Table_2.docx]

**Supplementary table 2** Changes of body weight during treatments

|  | NGT  (n=8) | T1D  (n=7) | INS  (n=7) | Lira  (n=8) | INS+Lira  (n=5) | p value |
| --- | --- | --- | --- | --- | --- | --- |
| 13W | 22.4±0.9 | 21.2±0.6* | 20.8±1.2 | 21.1±0.8 | 21.0±0.9 | 0.015 |
| 14W | 21.1±1.0 | 19.4±0.9* | 17.9±1.2# | 19.8±1.8† | 18.2±0.6‡ | <0.001 |
| 15W | 21.3±1.5 | 19.4±0.8* | 19.2±1.3 | 18.2±1.1 | 19.2±0.9 | <0.001 |
| 16W | 22.2±1.3 | 19.7±1.2* | 19.3±1.1 | 18.9±1.2 | 17.8±0.7#† | <0.001 |
| 17W | 22.9±1.1 | 20.5±1.3* | 20.2±1.6 | 19.5±1.6 | 18.2±1.1#† | <0.001 |
| 18W | 23.5±1.2 | 20.6±1.9* | 20.8±1.6 | 20.3±2.0 | 19.6±1.1 | <0.001 |
| 19W | 23.9±1.2 | 20.1±2.1* | 20.8±1.3 | 20.3±2.5 | 19.6±1.8 | 0.001 |
| 20W | 24.3±1.3 | 22.6±1.4* | 21.9±0.5 | 21.4±0.8 | 21.3±0.4 | <0.001 |
| 21W | 23.9±1.3 | 21.3±1.3* | 21.2±0.6 | 21.3±0.5 | 20.7±0.7 | <0.001 |

NGT: normal glucose tolerance group; T1D: type 1 diabetes group; INS: insulin treatment group; Lira: liraglutide treatment group; INS+Lira: insulin + liraglutide treatment group.

All data are expressed as mean ± SD;ANOVA was used for comparison between groups, and LSD method was used for multiple comparisons. p<0.05 was defined as statistically significant.

*:Compared to NGT

#:Compared to T1D

†:Compared to INS

‡:Compared to Lira
