# Supplementary material for: Liraglutide Inhibits Osteoclastogenesis and Improves Bone Loss by Downregulating Trem2 in Female Type 1 Diabetic Mice: Findings From Transcriptomics
Source: Front Endocrinol (Lausanne). 2021 Dec 15;12:763646. doi: 10.3389/fendo.2021.763646 (PMC8715718; doi:10.3389/fendo.2021.763646)
Supplement: Supplementary file 3 [file Table_3.docx]

**Supplementary table 3** Changes of blood glucose during treatments

|  | NGT  (n=8) | T1D  (n=7) | INS  (n=7) | Lira  (n=8) | INS+Lira  (n=5) | p value |
| --- | --- | --- | --- | --- | --- | --- |
| 13W | 8.1±1.4 | 17.6±3.6* | 22.9±6.4# | 19.3±3.8 | 24.2±7.8# | <0.001 |
| 14W | 6.4±0.8 | 20.4±6.7* | 26.1±3.6 | 16.1±6.9† | 20.9±9.6 | <0.001 |
| 15W | 7.4±1.3 | 21.5±7.0* | 26.1±5.0 | 18.4±8.1 | 19.2±10.6 | 0.001 |
| 16W | 8.5±0.7 | 24.2±7.5* | 25.6±3.2 | 18.8±8.4 | 22.9±9.5 | <0.001 |
| 17W | 8.1±0.9 | 23.0±6.9* | 22.9±4.9 | 15.9±8.1#† | 23.4±6.5‡ | <0.001 |
| 18W | 7.7±1.5 | 24.7±8.3* | 23.6±4.1 | 15.8±7.3#† | 22.9±9.8 | <0.001 |
| 19W | 8.4±0.9 | 19.5±5.8* | 20.6±6.6 | 17.4±5.5 | 24.2±6.5‡ | <0.001 |
| 20W | 6.7±0.6 | 14.8±5.5* | 17.5±3.2 | 8.4±2.3#† | 16.2±5.5‡ | <0.001 |
| 21W | 7.5±0.6 | 18.3±7.5* | 18.3±3.0 | 9.3±1.3#† | 16.8±5.5‡ | <0.001 |

NGT: normal glucose tolerance group; T1D: type 1 diabetes group; INS: insulin treatment group; Lira: liraglutide treatment group; INS+Lira: insulin + liraglutide treatment group.

All data are expressed as mean ± SD;ANOVA was used for comparison between groups, and LSD method was used for multiple comparisons. p<0.05 was defined as statistically significant.

*:Compared to NGT

#:Compared to T1D

†:Compared to INS

‡:Compared to Lira
