# Supplementary material for: Liraglutide Inhibits Osteoclastogenesis and Improves Bone Loss by Downregulating Trem2 in Female Type 1 Diabetic Mice: Findings From Transcriptomics
Source: Front Endocrinol (Lausanne). 2021 Dec 15;12:763646. doi: 10.3389/fendo.2021.763646 (PMC8715718; doi:10.3389/fendo.2021.763646)
Supplement: Supplementary file 4 [file Table_4.docx]

**Supplementary Table 4** Bone mineral density and microarchitectures of tibia measured by micro-CT

|  | NGT  (n=6) | T1D  (n=6) | INS  (n=6) | Lira  (n=6) | INS+Lira  (n=5) | p value |
| --- | --- | --- | --- | --- | --- | --- |
| Tb.vBMD(g/cm^3^) | 0.78±0.08 | 0.55±0.11* | 0.46±0.11 | 0.70±0.09#† | 0.60±0.09† | <0.001 |
| Ct.vBMD(g/cm^3^) | 3.72±0.18 | 3.03±0.22 ***** | 4.09±0.15# | 3.44±0.24#† | 3.56±0.55#† | <0.001 |
| BV/TV(%) | 25.0±6.0 | 18.0±6.6 | 13.0±2.9 | 23.0±6.7† | 21.0±9.7 | 0.029 |
| Tb.Th(mm) | 0.03±0.04 | 0.03±0.03 | 0.03±0.03 | 0.04±0.02 | 0.03±0.05 | 0.522 |
| Tb.N(1/mm) | 7.59±1.63 | 5.30±1.52* | 3.87±0.98 | 6.18±1.55† | 6.03±2.30† | 0.010 |
| Tb.Sp(mm) | 0.10±0.03 | 0.17±0.05* | 0.24±0.07# | 0.13±0.04† | 0.15±0.06† | 0.002 |
| Ct.Th(mm) | 0.13±0.01 | 0.12±0.01 | 0.13±0.01 | 0.14±0.01 | 0.13±0.02 | 0.057 |

NGT: normal glucose tolerance group; T1D: type 1 diabetes group; INS: insulin treatment group; Lira: liraglutide treatment group; INS+Lira: insulin + liraglutide treatment group.

Cortical volumetric bone mineral density (Ct.vBMD); Cortical bone thickness(Ct.Th); Trabecular volumetric bone mineral density (Tb.vBMD); Bone volume fraction (BV/TV); Trabecular thickness(Tb.Th); Trabecular number (Tb.N); Trabecular separation(Tb.Sp).

All data are expressed as mean ± SD;ANOVA was used for comparison between groups, and LSD method was used for multiple comparisons. p<0.05 was defined as statistically significant.

*:Compared to NGT

#:Compared to T1D

†:Compared to INS

‡:Compared to Lira
