# Supplementary material for: Liraglutide Inhibits Osteoclastogenesis and Improves Bone Loss by Downregulating Trem2 in Female Type 1 Diabetic Mice: Findings From Transcriptomics
Source: Front Endocrinol (Lausanne). 2021 Dec 15;12:763646. doi: 10.3389/fendo.2021.763646 (PMC8715718; doi:10.3389/fendo.2021.763646)
Supplement: Supplementary file 5 [file Table_5.docx]

**Supplementary Table 5** Bone histomorphometry of the femur

|  | NGT  (n=8) | T1D  (n=7) | INS  (n=7) | Lira  (n=8) | INS+Lira  (n=5) | p value |
| --- | --- | --- | --- | --- | --- | --- |
| BV/TV(%) | 6.55±1.44 | 6.80±3.37 | 6.64±2.93 | 6.88±2.73 | 8.61±3.83 | 0.796 |
| Tb.Th(mm) | 0.024±0.005 | 0.019±0.004 | 0.022±0.002 | 0.020±0.003 | 0.022±0.005 | 0.289 |
| Tb.N(1/mm) | 2.76±1.82 | 3.65±1.82 | 3.00±1.31 | 3.36±1.17 | 3.95±1.74 | 0.685 |
| Tb.Sp(mm) | 0.34±0.34 | 0.35±0.26 | 0.37±0.18 | 0.30±0.09 | 0.27±0.11 | 0.823 |
| Adipose.N(1/mm^2^) | 54.7±46.8 | 58.8±31.7 | 58.9±22.9 | 45.9±30.4 | 44.5±23.4 | 0.886 |

NGT: normal glucose tolerance group; T1D: type 1 diabetes group; INS: insulin treatment group; Lira: liraglutide treatment group; INS+Lira: insulin + liraglutide treatment group.

Bone volume fraction (BV/TV); Trabecular thickness(Tb.Th); Trabecular number (Tb.N); Trabecular separation(Tb.Sp); Adipose number in marrow(Adipose.N)

All data are expressed as mean ± SDs;ANOVA was used for comparison between groups, and LSD method was used for multiple comparisons. p<0.05 was defined as statistically significant.
