# Supplementary material for: Liraglutide Inhibits Osteoclastogenesis and Improves Bone Loss by Downregulating Trem2 in Female Type 1 Diabetic Mice: Findings From Transcriptomics
Source: Front Endocrinol (Lausanne). 2021 Dec 15;12:763646. doi: 10.3389/fendo.2021.763646 (PMC8715718; doi:10.3389/fendo.2021.763646)
Supplement: Supplementary file 6 [file Table_6.docx]

**Supplementary Table 6** Serum bone turnover markers and C-peptide

|  | NGT  (n=8) | T1D  (n=7) | INS  (n=7) | Lira  (n=8) | INS+Lira  (n=5) | p value |
| --- | --- | --- | --- | --- | --- | --- |
| P1NP(ng/ml) | 105.5±12.0 | 127.7±39.5 | 149.8±33.7 | 118.5±30.3 | 106.3±24.6 | 0.061 |
| CTX(pg/ml) | 97.8±39.1 | 120.0±21.8 | 96.9±26.6 | 78.7±13.6 | 133.4±45.3 | 0.07 |
| C-peptide(pg/ml) | 308.9±45.3 | 259.9±30.9 | 267.8±75.1 | 240.6±48.5 | 301.3±45.3 | 0.128 |

NGT: normal glucose tolerance group; T1D: type 1 diabetes group; INS: insulin treatment group; Lira: liraglutide treatment group; INS+Lira: insulin + liraglutide treatment group.

All data are expressed as mean ± SDs;ANOVA was used for comparison between groups, and LSD method was used for multiple comparisons. p<0.05 was defined as statistically significant.
